# Valero’s host index is useful in predicting radiation-induced trismus and osteoradionecrosis of the jaw risks in locally advanced nasopharyngeal carcinoma patients

**DOI:** 10.1186/s12885-023-11155-z

**Published:** 2023-07-12

**Authors:** Erkan Topkan, Efsun Somay, Busra Yilmaz, Berrin Pehlivan, Ugur Selek

**Affiliations:** 1 Department of Radiation Oncology, Medical Faculty, Baskent University, Adana, Turkey; 2 Department of Oral and Maxillofacial Surgery, Faculty of Dentistry, Baskent University, Ankara, Turkey; 3 Department of Oral and Maxillofacial Radiology, School of Dental Medicine, Bahcesehir University, Istanbul, Turkey; 4 Department of Radiation Oncology, Bahcesehir University, Istanbul, Turkey; 5 Department of Radiation Oncology, School of Medicine, Koc University, Istanbul, Turkey

**Keywords:** Nasopharyngeal carcinoma, Concurrent chemoradiotherapy, Host index, Trismus, Osteoradionecrosis

## Abstract

**Background:**

In the absence of previous research, we sought to assess the H-Index’s predictive significance for radiation-induced trismus (RIT) and osteoradionecrosis of the jaw (ORNJ) in patients with locally advanced nasopharyngeal carcinoma (LA-NPC) receiving concurrent chemoradiotherapy (C-CRT).

**Patients and methods:**

The research comprised 295 LA-NPC patients who had C-CRT and pre- and post-C-CRT oral exams between June 2010 and December 2021. The H-Index was calculated using neutrophils, monocytes, lymphocytes, hemoglobin, and albumin measurements obtained on the first day of C-CRT. Patients were divided into three and two H-index groups, respectively, based on previously established cutoff values (1.5 and 3.5) and the cutoff value determined by our receiver operating characteristic (ROC) curve analysis. The primary objective was the presence of any significant connections between pretreatment H-Index groups and post-C-CRT RIT and ORNJ rates.

**Results:**

RIT and ORNJ was diagnosed in 46 (15.6%) and 13 (7.8%) patients, respectively. The original H-Index grouping could only categorize RIT and ORNJ risks at a cutoff value of 3.5, with no significant differences in RIT and ORNJ rates between groups with H-Index 1.5 and 1.5 to 3.5 (P < 0.05 for each). The ideal H-Index cutoff for both RIT and ORNJ rates was found to be 5.5 in ROC curve analysis, which divided the entire research population into two groups: H-Index ≤ 5.5 (N = 195) and H-Index > 5.5 (N = 110). Intergroup comparisons revealed that patients in the H-Index > 5.5 group had significantly higher rates of either RIT (31.8% vs. 5.9%; P < 0.001) or ORNJ (17.3% vs. 2.2%; P < 0.001) than their H-Index ≤ 5.5 counterparts. The results of the multivariate analysis showed that H-Index > 5.5 was independently linked to significantly higher RIT (P < 0.001) and ORNJ (P < 0.001) rates.

**Conclusion:**

Pre-C-CRT H-Index > 5.5 is associated with significantly increased RIT and ORNJ rates in LA-NPC patients receiving definitive C-CRT.

## Introduction

A significant contributor to head and neck cancer morbidity and mortality are nasopharyngeal carcinomas (NPCs). Despite substantial breakthroughs in diagnostic imaging and mass screening approaches, 70–75% of all patients are identified with a locally advanced NPC (LA-NPC), presumably owing to the unique location and concealed nature of the disease [1, 2]. Because it significantly improves locoregional disease control and survival, definitive platinum-based concurrent chemoradiotherapy (C-CRT) has replaced radiation alone or sequential chemoradiotherapy regimens in these patients [3, 4]. Sadly, these advantages came at the expense of a marked rise in severe late complications, including radiation-induced trismus (RIT) and osteoradionecrosis of the jaw (ORNJ) in a sizeable percentage of patients.

Although RIT and ORNJ rates are decreasing owing to the advent of intensity-modulated radiation therapy (IMRT), they remain medical challenges to surmount because of their negative impacts on practically all patient-related quality of life (QOL) metrics [5–7]. The prevalence of RIT (5-65%) and ORNJ (4-20%) varies significantly depending on the tumor location, tumor extension to the masticatory apparatus or jaw, treatment-related variables, and definitions utilized [8, 9]. The traditional patient-, disease-, and treatment-related risk factors for RIT and ORNJ are commonly cited [10, 11], but the patient’s biological condition and accompanying biomarkers are generally overlooked. However, Somay et al. and Yilmaz et al. recently discovered that high pretreatment systemic immune-inflammation index (SII) values were associated with decreased short-term success after temporomandibular joint (TMJ) arthrocentesis and an increased need for tooth extractions after C-CRT, respectively [12, 13]. Moreover, Somay et al. recently reported that a low baseline hemoglobin-to-platelet ratio (HPR) in LA-NPC patients and a high neutrophil-to-lymphocyte ratio (NLR) in parotid gland cancer patients were significant predictors of RIT after C-CRT and RT, respectively [14, 15]. All these recent findings suggest the possibility of using biological markers that reflect a patient’s overall immunological and inflammatory status as reliable indicators of treatment efficacy and late toxicity rates.

Another recently discovered biological marker is the host index (H-Index), which was first investigated in oral cavity squamous cell carcinomas treated with primary surgery by Valero et al. in 2020 [16]. This novel comprehensive index was created by combining the routine neutrophil, monocyte, and lymphocyte counts as well as albumin and hemoglobin (Hb) levels from complete blood count and biochemistry tests. The findings of this study showed that patients with H-Index scores of 1.5 to 3.5 [hazard ratio (HR): 1.47] and 3.6 or higher (HR: 3.22) had a higher risk of death when compared to patients with an H-Index score of 1.4 or less. Later, these findings were validated for laryngeal, oropharyngeal, and hypopharyngeal cancer primaries, regardless of the extent of the disease or the type of therapy employed [17, 18]. Unfortunately, NPC patients were not included in these studies, and the common endpoint was survival results, and neither study examined the H-Index’s potential value in predicting treatment-related toxicities like the RIT and ORNJ.

Persistent systemic inflammation, the seventh hallmark of cancer [19], has been demonstrated to increase neutrophil, monocyte, and thrombocyte counts while decreasing lymphocyte levels [20, 21]. Albumin levels fall in hyper-inflammatory circumstances because C-reactive protein inhibits albumin production in hepatocytes [22]. Poor diet, which is frequent in cancer patients, may also cause low albumin levels [23]. Anemia or low Hb levels, indicators of tissue hypoxia, are also ubiquitously encountered in cancer patients, including those with LA-NPC [24]. Given the importance of these variables and associated cytokines in RIT and ORNJ genesis by inducing a favorable immune and inflammatory milieu, tissue hypoxia, vascular occlusion, and fibrotic tissue repair [25, 26], we hypothesized that the H-Index could reliably predict the risk of severe late toxicities in LA-NPC patients. Motivated by the accessible fundamental grounds, we planned to examine the novel H-Index for its utility in predicting the RIT and ORNJ in LA-NPC patients treated with definitive C-CRT.

## Patients and methods

### Data collection

**Fig. 1:**
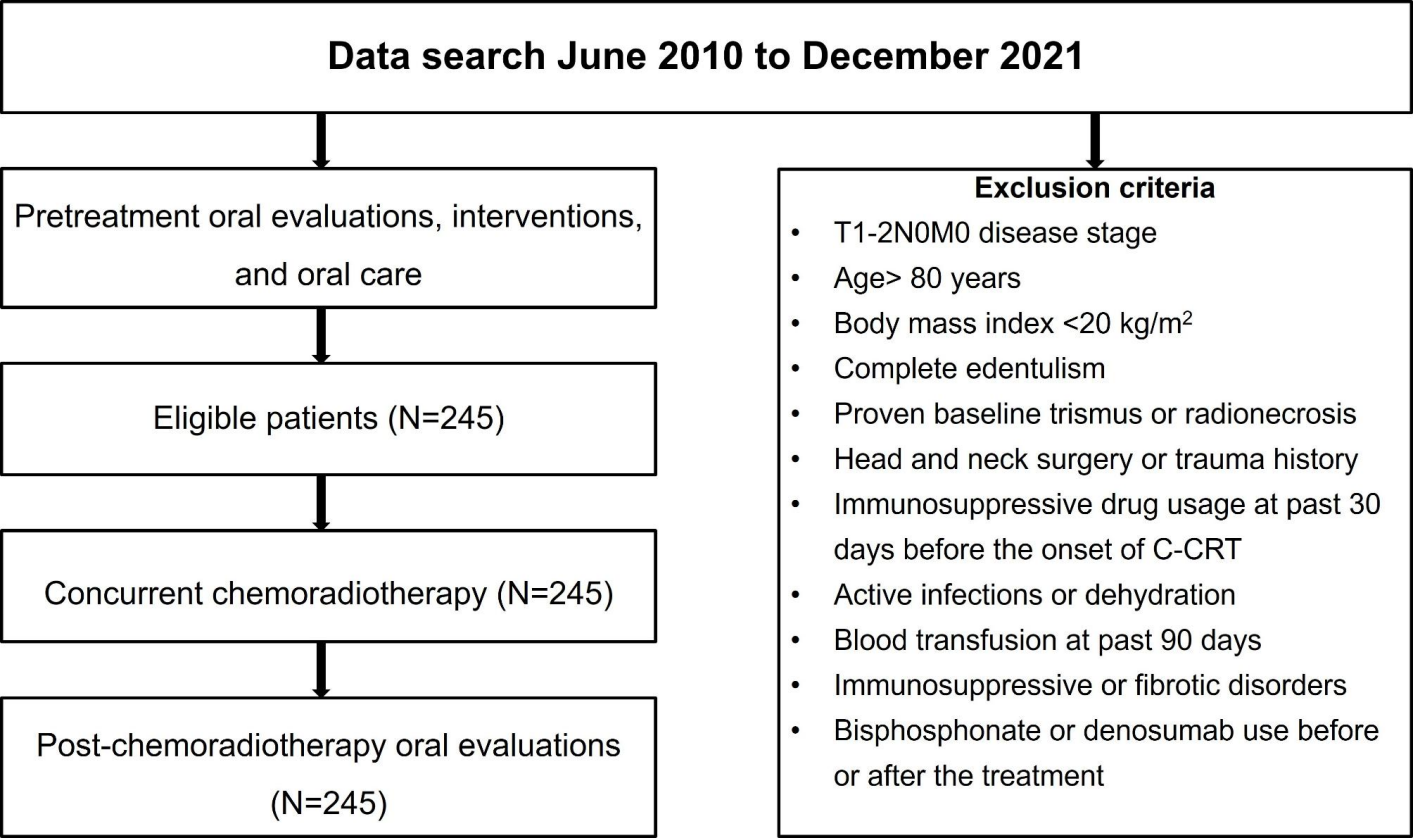
Flowchart diagram summarizing patient selection, baseline assessment, treatment, and follow-up information

The Departments of Radiation Oncology and Dentistry of Baskent University Medical Faculty collaborated in this retrospective study. All data were gathered through a retrospective review of the medical records of LA-NPC patients who underwent radical C-CRT and pre- and post-C-CRT oral examinations at our facility between June 2010 and December 2021. Patients with Eastern Cooperative Oncology Group (ECOG) performance of 0–1, type 2–3 squamous cell NPC, clinical/radiological proof of T1-2N2-3M0 or T3-4N0‐3M0 NPC after restaging per AJCC 8th ed., available baseline complete blood count and biochemistry tests, no chemotherapy/radiotherapy (RT) history, available baseline head and neck clinical examinations, chest computerized tomography (CT), brain magnetic resonance imaging (MRI), MRI of the nasopharynx and the neck, and fluorodeoxyglucose-positron emission CT (PET-CT) scans, available RT and chemotherapy charts, no evidence of masticatory apparatus disorders including the temporomandibular joint, and available records of pre- and post-treatment oral and ear-nose-throat examinations, and follow-up records of radiological examinations were deemed eligible for the study (Fig. 1).

### Ethics and consent

This retrospective study protocol adhered to the official rules of the Declaration of Helsinki and its amendments and was approved by the institutional Ethics and Science Committee of Baskent University Medical Faculty before collecting patient data. As mandated by our institutional standards, all patients gave their written informed consent prior to the start of C-CRT, either directly or through legally appointed representatives, for the collection and analysis of blood samples and pathologic specimens, as well as for the academic presentation and publication of results.

### Treatment protocol

All LA-NPC patients received simultaneous integrated boost intensity-modulated RT (SIB-IMRT) under our institutional standards, as documented elsewhere [13]. Each target volume was determined using pretreatment co-registered computed tomography (CT), 18-FDG-PET-CT, and/or MRI scans of the implicated NPC primary and the entire neck. The target volumes and associated RT doses during the treatment period were established using institutional standards and readily accessible guidelines [13]. For planning target volumes (PTV) of high-, intermediate-, and low-risk, respectively, the total instructed doses were 70 Gy, 59.4 Gy, and 54 Gy, delivered in 33 daily fractions. On days 1, 22, and 43, RT was combined with three cycles of concurrent chemotherapy that included cisplatin and 5-fluorouracil. After C-CRT, patients were advised to complete two cycles of the same chemotherapy regimen used during the C-CRT phase of their treatment as adjuvant therapy, provided that it was tolerable for them. All patients received supportive care measures when deemed necessary.

### Baseline and follow-up RIT and ORNJ assessments

Whether or not they had symptoms, every patient underwent a thorough oral examination before C-CRT, as recommended by the American Dental Association and the US Food and Drug Administration [27]. An experienced surgeon (ES) and an oral and maxillofacial radiologist (BY) evaluated the oral cavity and associated structures clinically and radiologically in all cases. In accordance with our institutional norms, panoramic radiographs were used for radiographic oral and dental examinations on all patients. The same Veraviewepocs 2D X-ray machine (J Morita, Kyoto, Japan) was used for all digital panoramic radiographs, and the patients were positioned following the manufacturer’s instructions. The exposure times were 70 kVp, 10 mA, and 9 s.

In this study, RIT was defined as having a maximum mouth opening (MMO) of ≤ 35 mm in accordance with the standards previously established by Dijkstra et al. [28]. Because of its proven measurement precision and ease of application, Therabite® (Atos Medical AB, Hörby, Sweden) was chosen to quantify MMOs [29]. Each patient was positioned with their head parallel to the Frankfurt horizontal plane, facing forward. The patients were instructed to open their mouths as wide as possible while wearing the Therabite® motion scale to measure the distance between the lower edge of one of the upper central incisors and the upper edge of one of the corresponding mandibular central incisors. The mean MMO was calculated as the arithmetical average of three successive measures per session. The post-C-CRT MMO measurements were collected for each patient at 1, 3, 6, 9, 12, 18, and 24 months to assess RIT status using the identical protocol employed for the baseline measurements. Subsequently, these measurements were conducted during each scheduled biannual follow-up interval or more frequently in instances of suspicion.

The ORNJ status was determined based on radiological evidence of ORNJ with intact mucosa and clinical and radiological ORNJ diagnostic criteria [30]. Accordingly, ORNJ was clinically defined as irradiated necrotic bone tissue that failed to heal for a period of 3 months without any signs of tumor progression or metastasis [30]. To ensure a timely diagnosis of ORNJ, each patient received scheduled clinical and radiological examinations at 1, 3, 6, 9, 12, 18, and 24 months after C-CRT completion. Then, the identical methodology was utilized in each predetermined semiannual follow-up period, or more often if ORNJ was suspected. For ORNJ staging, Notani’s classification—which takes into account small bone alterations and anatomical boundaries of lesions—was applied [31].

### Baseline host index (H-Index) evaluation

Pretreatment complete blood count and biochemistry test results acquired on the first day of C-CRT were utilized to calculate the H-Index. We calculated the pre-CCRTH-Index using Valero and colleagues’ original following formula [16]:

$$\eqalign{& Host\,Index =\cr & \frac{{Neutrophils \times Monocytes}}{{Lymphocytes \times Hemoglobin \times Albumin }} \times 100 }$$

### Statistical methods

The primary objective of this retrospective cohort study was to investigate any potential correlations between pre-CCRT H-Index groups and post-CCRT RIT and ORNJ incidences. We employed two distinct approaches to achieve this goal: First, patients were divided into three groups based on their H-Index scores using Valero’s original 1.5 and 3.5 cutoff values [16]. And second, we used receiver operating characteristic (ROC) curve analysis to determine the ideal cutoffs that, if they exist, could split the entire research cohort into two subgroups with different RIT and ORNJ outcomes, respectively. While categorical variables were expressed with percent frequency distributions, continuous variables were described with medians and ranges. To compare frequency distributions of the desired factor, such as H-Index scores, according to the different clinical variables, the Chi-square test, Student’s t-test, Pearson’s exact test, ANOVA, or Spearman’s correlation estimates were used as indicated. Only factors that had been found to be significant in the univariate analysis were included in the multivariate analysis. Every P value was two-sided, and a value of < 0.05 was considered significant. In order to reduce the random false-positive results from simultaneously performing multiple subgroup analyses (≥ 3 subgroups), such as Valero’s H-Index groups, the treatment weights were multiplicity corrected using Bonferroni corrections, and the resulting P-values were employed to determine the significance level. Statistical Package for the Social Sciences (SPSS) version 26 was used for all statistical analyses.

## Results

### Patient and disease characteristics

A total of 372 stage LA-NPC patients were distinguished from the records, but 77 of them were disqualified for the following reasons: receiving upfront induction chemotherapy (N = 58), refusing chemotherapy (N = 12), and having trismus (N = 7). Thus, 295 patients qualified for this study. Table 1 depicts the patients and disease characteristics of the entire population. The cohort’s median age was 56 years (interquartile range: 18–78 years), and 23.7% of the participants (N = 70) were over 70. Probably reflecting the poor oral care habits, all patients underwent dental extraction(s), with a median interval to C-CRT of 16 days (range 10–24 days).

**Table 1:** Pretreatment patient and disease characteristics for the entire study population and according to Host Index groups

Factor	All patients (N = 295)	H-Index ≤ 5.5 (N = 185)	H-Index > 5.5 (N = 110)	P-value
Median age, years (range)	56 (18–78)	57 (18–77)	55 (23–77)	0.76
Age group, N (%) ≤ 70 years > 70 years	225 (76.3) 70 (23.7)	140 (75.6) 45 (24.4)	85 (77.2) 25 (22.8)	0.81
Gender, N (%) Female Male	97 (32.9) 198 (67.1)	63 (34.0) 122 (66.0)	34 (30.9) 76 (69.1)	0.61
Body mass index; kg/m^2^ (range)	23.2 (20.1–29.4)	23.7 (20.6–29.1)	22.9 (20.1–29.4)	0.52
Smoking status, N (%) No Yes	105 (35.6) 190 (64.4)	71 (38.4) 114 (61.6)	34 (30.9) 76 (69.1)	0.21
Alcohol consumption, N (%) No Yes	172 (58.3) 123 (41.7)	104 (56.2) 81 (43.8)	68 (61.8) 42 (38.2)	0.39
Pre-C-CRT dental extraction, N (%) No Yes	0 (0.0) 295 (100.0)	0 (0.0) 185 (100.0)	0 (0.0) 110 (100.0)	1.0
Dental extraction to C-CRT interval, days (range)	16 (10–24)	17 (11–24)	15 (10–23)	0.58
Median pre-C-CRT MMO, mm (range)	41.2 (37.5–46.8)	41.4 (37.8–46.4)	41.0 (37.5–46.8)	0.74
Pre-C-CRT MMO group, N (%) ≤ 41.2 mm > 41.2 mm	148 (50.2) 147 (49.8)	89 (48.1) 96 (51.9)	59 (53.6) 51 (46.4)	0.40
T-stage group, N (%) 1–2 3–4	76 (25.7) 219 (74.3)	49 (26.5) 136 (73.5)	27 (24.5) 83 (75.5)	0.79
N-stage, N (%) 0–1 2–3	65 (22.0) 230 (78.0)	45 (24.3) 140 (75.7)	20 (18.2) 90 (81.8)	0.22

**Abbreviations**: H-Index: Host Index; C-CRT: Concurrent chemoradiotherapy; MMO: Maximum mouth opening; T-stage: Tumor stage; N-stage: Nodal stage

### Treatment and dosimetric characteristics

Of the 295 eligible patients, 234 (79.3%) and 216 (73.2%) received 2–3 cycles of concurrent chemotherapy and 1–2 cycles of adjuvant chemotherapy, respectively (Table 2). Significant weight loss (SWL), defined as a loss of > 5% of body weight, was observed in 112 (38.0%) patients during C-CRT. Following C-CRT, 230 patients (78.0%) had additional tooth extractions at a median follow-up of 58.4 months (range: 4.7–126.8 months). The mean masticatory apparatus dose (MAD), mean mandibular dose (MMD), and mean maximum mandibular point dose (MMPD) were 38.9 Gy (range: 21.3–76.4 Gy), 35.9 Gy (10.4–51.3 Gy), and 54.4 Gy (32.7–78.5 Gy), respectively, for the whole study population (Table 2).

**Table 2:** Treatment characteristics, dosimetric results, and clinical outcomes for the entire study population and according to Host Index groups

Factor	All patients (N = 295)	H-Index ≤ 5.5 (N = 185)	H-Index > 5.5 (N = 110)	P-value
Concurrent chemotherapy cycles, N (%) 1 2–3	61 (20.7) 234 (79.3)	35 (18.9) 150 (81.1)	26 (23.6) 84 (76.4)	0.33
Adjuvant chemotherapy cycles, N (%) 0 1–2	79 (26.8) 216 (73.2)	46 (24.9) 139 (75.1)	33 (30.0) 77 (70.0)	0.42
Significant weight loss during C-CRT, N (%) No Yes	183 (62.0) 112 (38.0)	126 (68.1) 59 (31.9)	57 (51.8) 53 (48.2)	0.004
Post-C-CRT dental extraction No Yes	65 (22.0) 230 (78.0)	44 (23.8) 141 (76.2)	21 (19.1) 89 (80.9)	0.54
Median post-C-CRT MMO, mm (range)	38.3 (25.9–44.0)	38.4 (28.3–43.0)	38.0 (25.9–44.0)	0.81
Mean masticatory apparatus dose, Gy (range)	38.9 (21.3–76.4)	37.2 (23.7–75.8)	40.1 (21.3–76.4)	0.38
Mean MAD dose, N (%) < 48.5 Gy ≥ 48.5 Gy	158 (53.6) 137 (46.4)	98 (52.9) 87 (47.1)	60 (54.5) 50 (45.5)	0.83
Median MMPD; Gy (range)	54.4 (32.7–78.5)	56.3 (32.7–77.1)	53.2 (33.4–78.5)	0.26
MMD, Gy (range)	35.9 (10.4–51.3)	35.1 (10.4–50.2)	36.8 (10.8–51.3)	0.79
MMD group, N (%) < 36.2 Gy > 36.2 Gy	162 (54.9) 133 (45.1)	96 (51.9) 89 (48.1)	66 (60.0) 44 (40.0)	0.22
Mandibular V_59 Gy_ group, N (%) < 32% ≥ 32%	203 (68.8) 92 (31.2)	121 (65.4) 64 (34.6)	82 (74.5) 28 (25.5	0.23
Median C-CRT to RIT interval, mo. (range)	10 (6–18)	11 (7–18)	9 (6–17)	0.67
RIT, N (%) Absent Present	249 (84.4) 46 (15.6)	174 (94.1) 11 (5.9)	75 (78.2) 35 (31.8)	< 0.001
Median C-CRT to ORNJ interval, mo. (range)	19 (15–34)	22 (16–34)	18 (15–32)	0.22
ORNJ, N (%) Absent Present	23 (7.8) 272 (92.2)	4 (2.2) 181 (97.8)	19 (17.3) 91 (82.7)	< 0.001
Notani’s ORNJ stage 0 1 2	272 (92.2) 16 (5.4) 7 (2.4)	181 (97.8) 3 (1.6) 1 (0.6)	91 (82.7) 13 (11.8) 6 (5.5)	< 0.001

**Abbreviations**: H-Index: Host Index; C-CRT: Concurrent chemoriadiotherapy; MMO: Maximum mouth opening; MAD: Masticatory apparatus dose; MMPD: Maximum mandibular point dose; MMD: Mean mandibular dose; V_59 Gy_: Volume receiving 59 Gy or higher; RIT: Radiation-induced trismus; ORNJ: Osteoradionecrosis of the jaw

#### Treatment outcomes

**Fig. 2:**
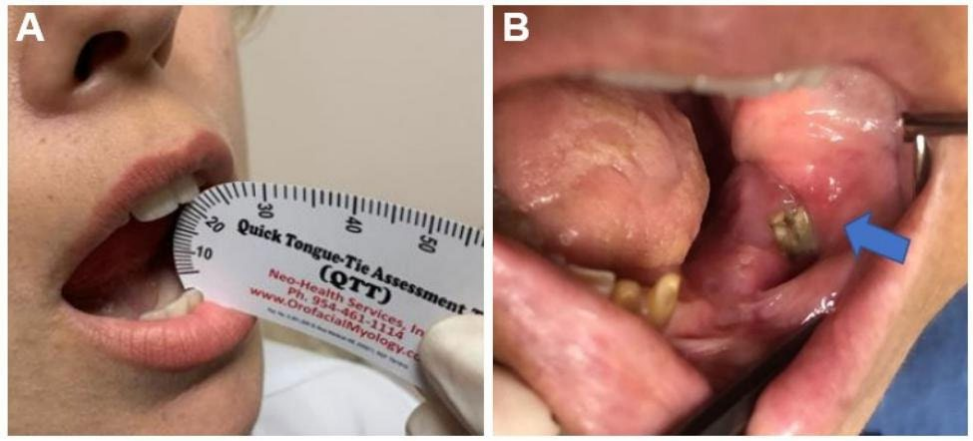
Clinical photographs of the patients. **(A)** A post-chemoradiotherapy trismusnt and **(B)** post-chemoradiotherapy osteoradionecrosis of the left jaw

During this research, MMO declined from 41.2 mm (37.5–46.8 mm) to 38.3 mm (24.9–44.0 mm), a reduction of 7.0%. With a median C-CRT to RIT interval of 10 months (range: 6–18 months), 46 (15.6%) individuals were diagnosed with RIT (Fig. 2). Twenty-three (7.8%) individuals had ORNJ after C-CRT (Fig. 2), with a median duration of 19 months (range: 15–34 months). Notani’s ORNJ staging [31] identified 16 (5.4%) and 7 (2.4%) patients as stages I and II, respectively. There were no patients identified with both RIT and ORNJ. Our subsequent analyses based on the ROC curve did not identify a critical time cutoff at which the incidence of RIT or ORNJ significantly increased beyond the first incident cases, which occurred at 6 months for RIT and 15 months for ORNJ. Particularly, the lack of ORNJ after 34 months may be linked to our stringent standards, which require the preservation of teeth during the follow-up period unless extraction is deemed necessary.

We examined ROC curve analysis cutoff points for continuous factors such as age, pre-C-CRT MMO, mean MAD, mean MMPD, MMD, and H-Index that may affect RIT and ORNJ clinical results (Table 2). Our analysis found that the pre-C-CRT MMO and mean MAD critical cutoffs for RIT incidence were 41.2 mm and 48.5 Gy, respectively. In the same way, the relevant cutoffs for MMD and mandibular V59 Gy that interact with ORNJ rates were found to be 36.2 Gy and 32%, respectively.

#### RIT and ORNJ results based on Valero’s H-Index

**Fig. 3:**
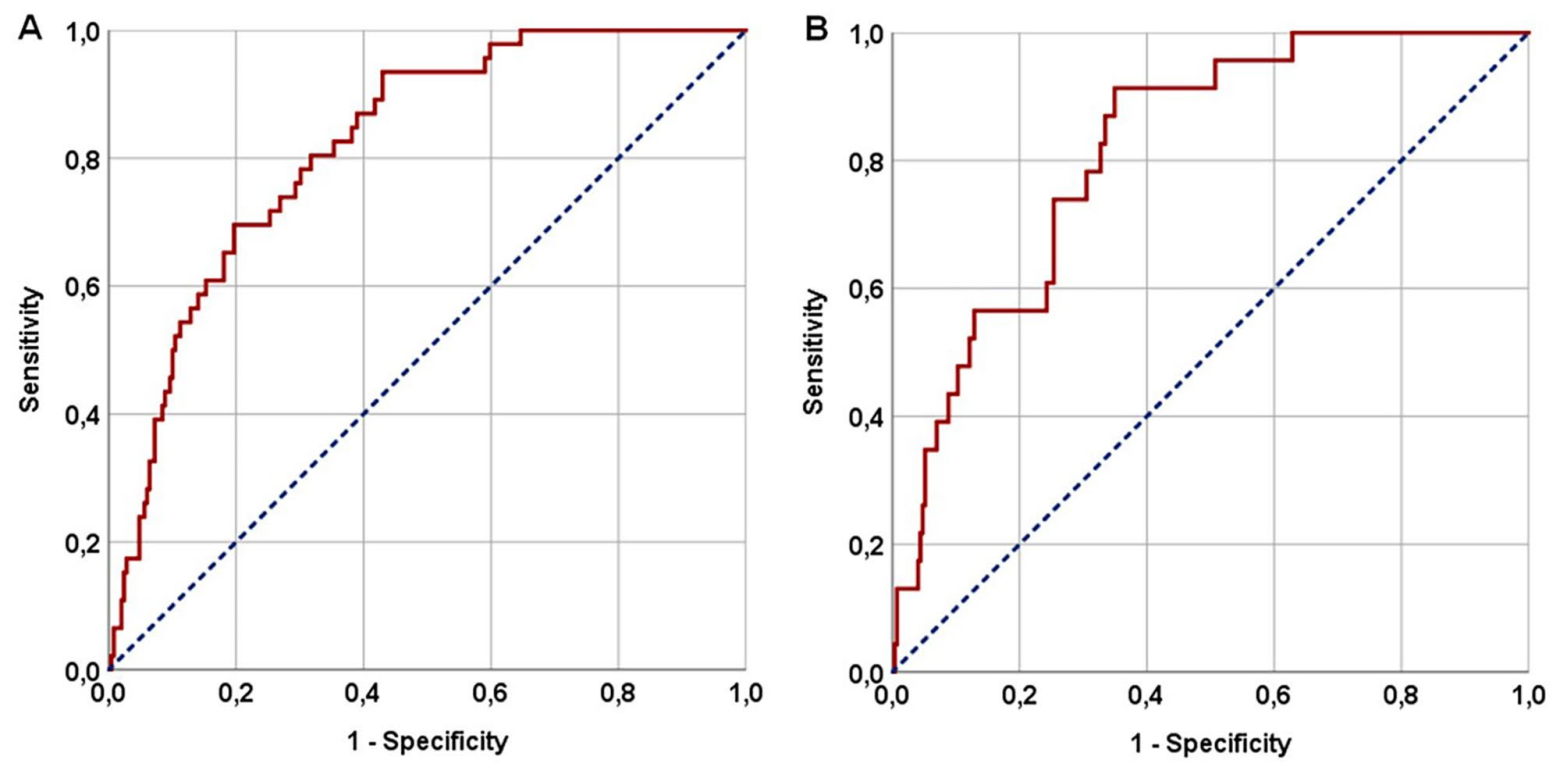
The results of receiver operating characteristic curve analyses: **(A)** Radiation-induced trismus, and **(B)** Osteoradionecrosis of the jaw

**Fig. 4:**
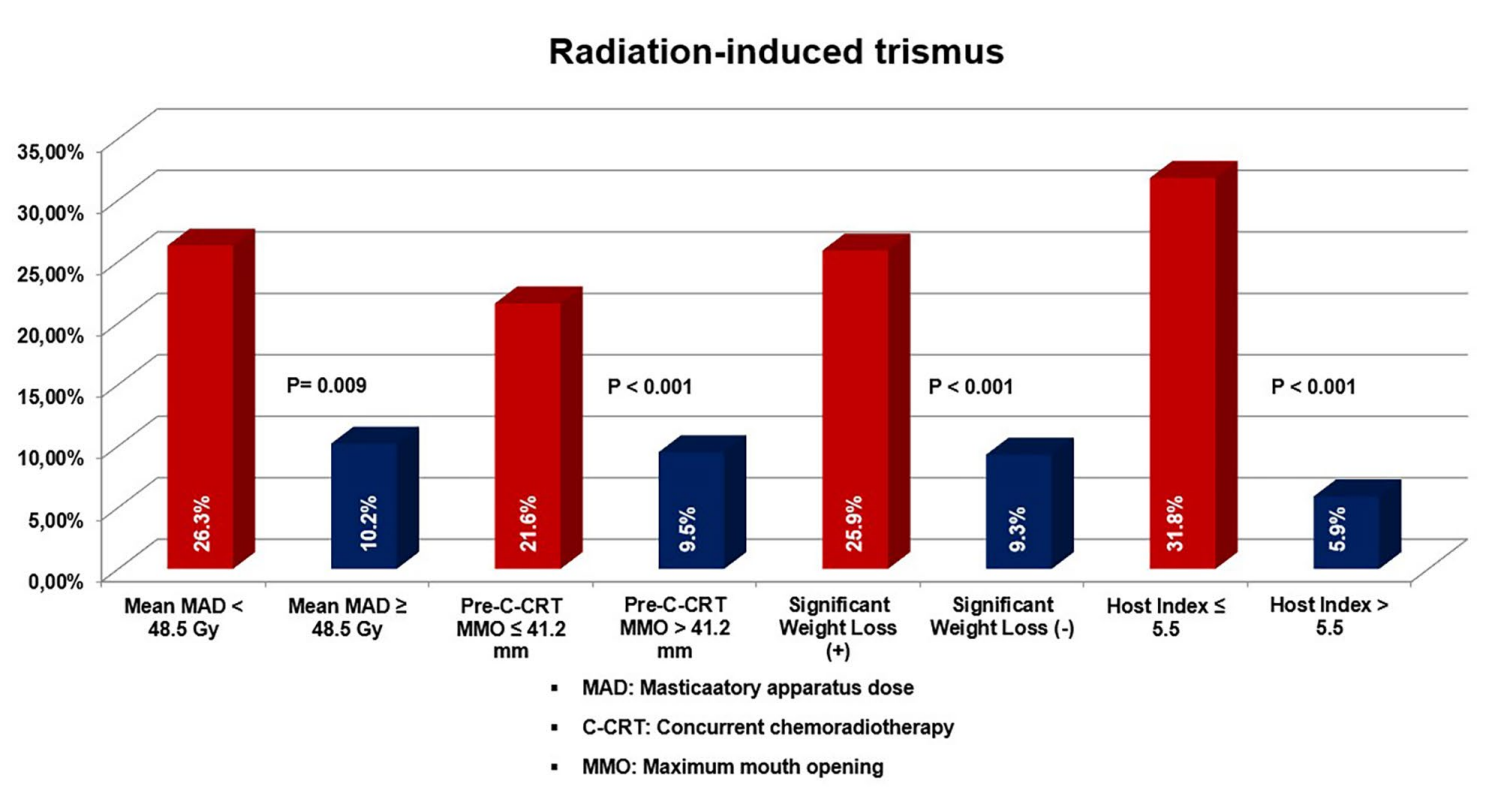
The bar chart depicts the rates of radiation-induced trismus according to the factors that showed independent significance in multivariate analysis

**Fig. 5:**
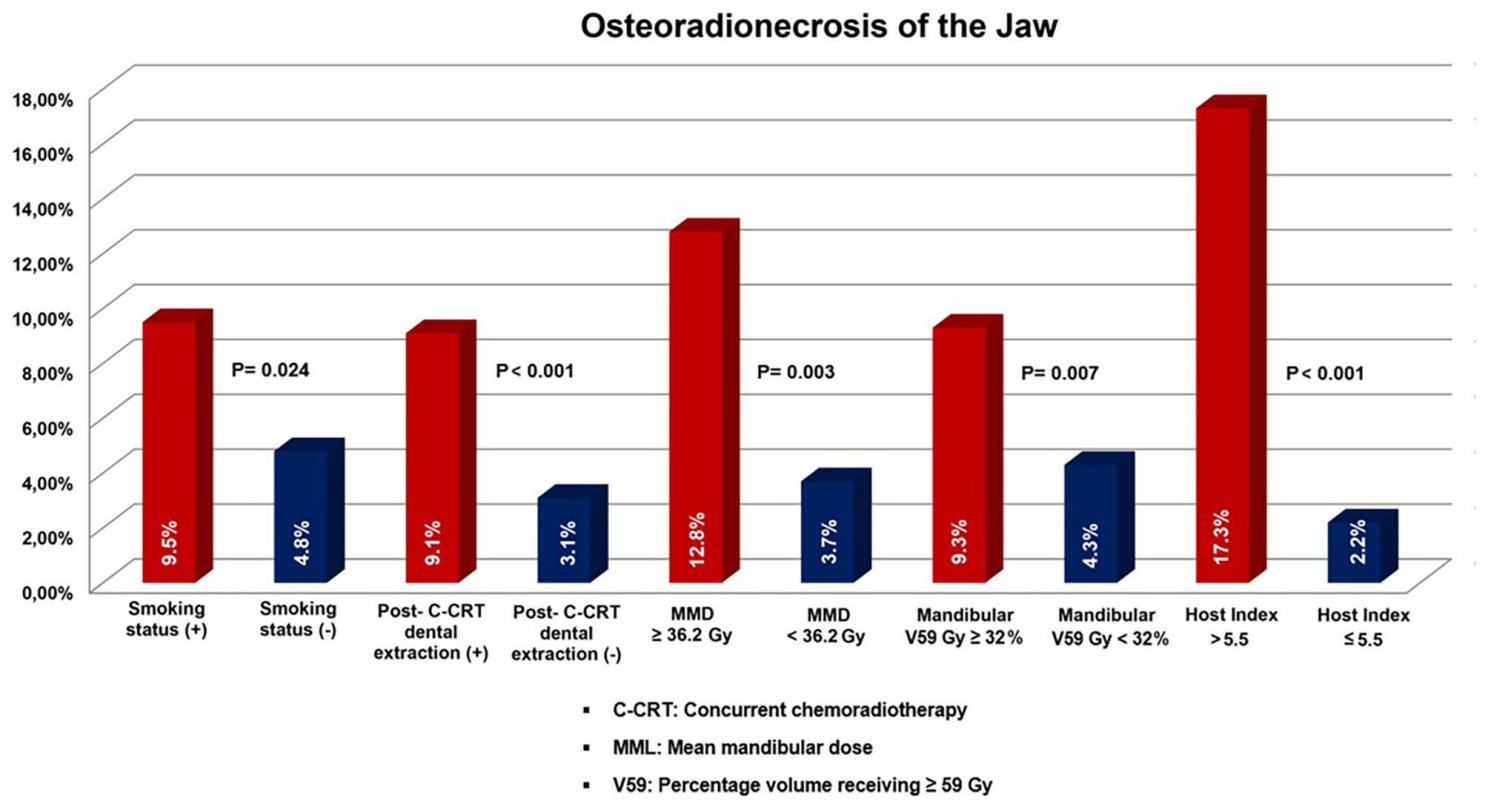
The bar chart depicts the rates of osteoradionecrosis of the jaw according to the factors that showed independent significance in multivariate analysis

Valero’s H-Index classification approach placed 36, 68, and 191 patients in Group 1 (H-Index < 1.5), Group 2 (H-Index between 1.5 and 3.5), and Group 3 (H-Index > 3.5). RIT instances were 0 (0%), 1 (1.4%), and 45 (23.6%) in groups 1, 2, and 3. ORNJ diagnoses were made in 1 (2.8%), 2 (2.9%), and 20 (10.5%) of the respective H-Index groups. The RIT (P = 0.89) and ORNJ (P = 0.97) rates in groups 1 and 2 were not significantly different (RIT = 0.89, ORNJ = 0.97). However, Group 3 patients had substantially higher RIT and ORNJ rates than Group 1 and Group 2 patients (P < 0.001 for each comparison) (Fig. 1). Because Valero’s H-Index failed to differentiate between Groups 1 and 2, we performed ROC curve analyses to find new cutoffs that might better distinguish RIT and ORNJ results. According to ROC curve analyses (Fig. 3), the respective H-Index cutoffs interacting with the RIT and ORNJ rates after C-CRT were 5.46 [Area under the curve (AUC): 82.3%; sensitivity: 79.4%; specificity: 76.8%; Youden index: 0.62] and 5.58 (AUC: 81.6%; sensitivity: 77.2%; specificity: 75.4%; Youden index: 0.59). Since the cutoffs were practically comparable, we picked a rounded 5.5 as the optimal cutoff to divide patients into two groups: H-Index 1: H-Index < 5.5 (N = 185), and H-Index 2: H-Index ≥ 5.5 (N = 110). Comparative analysis revealed that RIT (31.8% vs. 5.9% for H-Index < 5.5; P < 0.001) and ORNJ (17.3% vs. 2.2% for H-Index < 5.5; P < 0.001) rates were both markedly higher in the H-Index ≥ 5.5 patient group (Table 2, and Figs. 4 and 5). As indicated in Table 2, the substantial difference between H-Index groups maintained its significance regarding ORNJ stages (P < 0.001 for each comparison). Intriguingly, 29 (25.9%) of 112 SWL patients and 17 (9.3%) of 183 non-SWL patients developed RIT following C-CRT (P = 0.003), suggesting a substantial relationship between these characteristics.

#### Univariate and multivariate results

As indicated in Table 3, univariate analyses that included all probable covariates showed that smoking history (P = 0.039), presence of SWL during C-CRT (P < 0.001), a pre-C-CRT MMO of < 41.2 mm, a mean MAD dosage of > 48.5 Gy, and an H-Index > 5.5 were related with elevated incidences of RIT. All factors maintained their significance on RIT rates in the multivariate analysis (P < 0.05 for each), with the exception of smoking history (P = 0.14) (Fig. 2). Similarly, smoking history (P = 0.024), presence of post-CCRT dental extractions (P < 0.001), an MMD ≥ 36.2 Gy (P = 0.003), a mandibular V59 Gy ≥ 32% (P = 0.007), and an H-Index > 5.5 (P < 0.001) appeared to be significant associates of increased ORNJ rates in univariate analysis, all of which maintained their independent significance in multivariate analysis (P < 0.05 for each) (Table 3, and Figs. 4 and 5).

**Table 3:** Results of univariate and multivariate analyses for potential clinical and dosimetric factors that may interact with radiation-induced trismus and osteoradionecrosis of the jaw outcomes

	RIT			ORNJ		
Factor	Univariate P-value	Multivariate P-value	HR (95% CI)	Univariate P-value	Multivariate P-value	HR (95% CI)
Age group (≤ 70 years vs. >70 years)	0.82	-	1.03 (0.93–1.13)	0.86	-	1.06 (0.91–1.21)
Gender (male vs. female)	0.74	-	1.07 (0.91–1.23)	0.83	-	1.14 (0.83–1.45)
Smoking status (no vs. yes)	0.039	0.14	1.38 (0.87–1.89)	0.024	0.037	1.63 (1.29–1.97)
Alcohol consumption (no vs. yes)	0.24	-	1.12 (0.84–1.40)	0.38	-	1.12 (0.89–1.35)
T-stage group (1–2 vs. 3–4)	0.39	-	1.23 (0.92–1.54)	0.29	-	1.09 (0.93–1.26)
N-stage (0–1 vs. 2–3)	0.27	-	1.16 (0.94–1.38)	0.23	-	1.11 (0.95–1.27)
SWL (no vs. yes)	< 0.001	0.002	2.76 (2.28–3.24)	0.42	-	1.42 (0.96–1.88)
Pre-C-CRT MMO group (≤ 41.2 mm vs. >41.2 mm)	< 0.001	< 0.001	2.49 (1.56–3.32)	-	-	-
Post-C-CRT dental extraction (no vs. yes)	-	-	-	< 0.001	< 0.001	4.81 (3.26–6.36)
Concurrent chemotherapy cycles (1 vs. 2–3)	0.56	-	1.10 (0.97–1.23)	0.32	-	1.07 (0.88–1.26)
Adjuvant chemotherapy cycles (0 vs. 1–2)	0.34	-	1.08 (0.90–1.26)	0.67	-	1.04 (0.92–1.14)
Mean MAD dose (< 48.5 Gy vs. ≥48.5 Gy)	0.009	0.018	1.85 (1.41–2.29)	-	-	-
MMD group < 36.2 Gy vs. ≥36.2 Gy)	-	-	-	0.003	0.011	2.32 (2.01–2.63)
Mandibular V59 Gy group (< 32% vs. ≥ 32%)	-	-	-	0.007	0.009	2.36 (1.81–2.91)
H-Index groups (≤ 5.5 vs. > 5.5)	< 0.001	< 0.001	5.55 (4.17–6.83	< 0.001	< 0.001	7.24 (5.86–8.62)

**Note**: The reference parameters are denoted by the first values in parenthesis

**Abbreviations**: RIT: Radiation-induced trismus; ORNJ: Osteoradionecrosis of the jaw; T-stage: Tumor stage; N-stage: Nodal stage; SWL: Significant weight loss (> %5%): C-CRT: Concurrent chemoradiotherapy; MMO: Maximum mouth opening; MAD: Masticatory apparatus dose; MMD: Mean mandibular dose; V_59 Gy_: Volume receiving 59 Gy or higher; H-Index: Host Index

## Discussion

The H-Index was tested for its ability to predict RIT and ORNJ rates in conclusively treated LA-NPC patients. In addition to confirming conventional risk factors, our findings showed that the ROC curve analysis-derived 5.5 cutoff value and the 3.5 cutoff value proposed by Valero and colleagues were both efficient in independently classifying these patients into two distinct risk groups for RIT and ORNJ rates [16]. SWL during C-CRT was shown to be an additional independent predictor of greater RIT and ORNJ rates in our study. If confirmed by further research, these findings support the hypothesis that baseline quantities of immune cells, their secretory products, hypoxia, and nutritional state all play crucial roles in the genesis and advance of severe radiation-induced late toxicities.

In our study, 46 (15.6%) patients were diagnosed with RIT and 23 (7.8%) with ORNJ after C-CRT, which is consistent with the associated literature. The reported trismus rate in the MD Anderson Head and Neck Cancer Symptom Working Group study published in 2020, for example, was 29% [32]. Even though the 7.8% ORNJ rate presented here appears to be slightly higher than the < 5% references, it is compatible with earlier IMRT studies. ORNJ rates in two IMRT trials reported by Tsai et al. [33] and Maesschalck et al. [34] were 6.0% and 10.2%, respectively. Numerous conventional disease-, patient-, and dosimetry-related factors have been linked to increased RIT and ORNJ rates in various head and neck tumors treated with RT or C-CRT, including LA-NPC [35, 36]. These factors include tumors of the oral cavity and oropharynx, larger tumor sizes, higher T- and N stages, larger tumor sizes, previous surgery, the proximity of the primary tumor or involved lymph nodes to the masticatory apparatus and mandible, dental extractions before or after treatment, the presence of TMJ disorders, the use of concurrent chemotherapy, higher prescribed tumor doses, higher mean or median doses to the masticatory apparatus and mandible, and larger volumes of the masticatory muscles, joints, or mandibular bone receiving doses above a specified dose level [35, 36]. In this regard, the current multivariate results confirmed the independent predictive significance of a pre-C-CRT MMO of ≤ 41.2 mm and a mean MAD dose of ≥ 48.5 Gy for higher RIT (P 0.05 for each) and the presence of smoking history, post-C-CRT dental extractions, an MMD of ≥ 36.2 Gy, and a mandibular V59 Gy of ≥ 32% (P 0.05 for each) for higher ORNJ rates.

Our study’s first notable discovery was the demonstration of a significant relationship between SWL during C-CRT and a higher rate of RIT (25.9% vs. 9.3%; HR: 2.76; P = 0.002). Before or during C-CRT, patients with LA-NPC may exhibit SWL and nutritional deficiencies, which is a recognized prognostic indicator for these patients [37, 38]. Shen et al. [37] and Zeng et al. [38] found that any WL above the SWL thresholds of 5% and 4.6% was associated with significantly lower disease-free-, locoregional progression-free-, and overall survival rates in 2,433 and 606 curatively treated NPC patients, respectively, even after IMRT. Although RIT is often reported as one of the major causes of SWL in patients with head and neck cancer [39], SWL has never been investigated as a cause of RIT. Despite the complex link between RIT and SWL, our findings are credible since 29 (25.9%) of 112 SWL patients and only 17 (9.3%) of 183 non-SWL patients exhibited RIT following C-CRT (P = 0.001). SWL during C-CRT may also be a sign of weakened immunity, persistent inflammation, cancer development, and/or related pre-cachexia or cachexia, all of which may contribute to RIT besides serving as prognostic factors in such patients. This logical consequence is relevant considering the critical roles performed by inappropriate immunity, persistent inflammation, cachexia-related muscle loss, and enhanced fibrotic repair processes in all components of the masticatory apparatus during RIT pathogenesis [25].

In addition to its previously demonstrated utility in prognostic stratification of the oral cavity, oropharynx, hypopharynx, and larynx cancers [16–18], we established a first for LA-NPC literature by showing that the risk for (31.8% vs. 5.9% for H-Index < 5.5; P < 0.001) and ORNJ (17.3% vs. 2.2% for H-Index < 5.5) after C-CRT rises with increasing H-Index value. The H-Index potentially offers a host-related biomarker for categorizing survival outcomes or toxicity rates after a given oncological therapy by integrating immunological, inflammatory, nutritional, and oxygenation status surrogates like albumin and Hb. Although we were unable to demonstrate a significant utility for H-Index in terms of its predictive capabilities for three distinct RIT and ORNJ groups using Valero’s 1.5 and 3.5 cutoffs [16], we confirmed that the 3.5 cutoff was successfully able to stratify these patients into two significantly different groups concerning RIT (23.6% vs. 0.96% for H-Index < 3.5; P < 0.001) and ORNJ (10.5% vs. 2.9% for H-Index < 3.5; P < 0.001). Further, we clinched that 5.5 was the ideal H-Index cutoff in ROC curve analysis to divide these patients into two groups with significantly different risks for RIT (31.8 vs. 5.9% for H-Index < 5.5; P < 0.001) and ORNJ (17.3 vs. 2.2% for H-Index < 5.5; P < 0.001) after definitive C-CRT. Although the specific cause of this cutoff variation is unknown, it might be attributable to variances in the endpoints, tumor locales, tumor stages, and treatment modalities used here and elsewhere [16–18]. Further substantiating the relevance of this statement, an H-Index of 8.37 was found to be the ideal cut-off to distinguish the group of surgically treated laryngeal cancer patients with a higher risk of both recurrence/death (HR: 2.82) and only death (HR: 2.22) in the study reported by Boscolo-Rizzo et al. [17].

The exact pathophysiological mechanisms underlying the association between a high pre-C-CRT H-Index and noticeably elevated RIT and ORNJ rates remain unknown. However, valuing the distinct immune and inflammatory functions of neutrophils, monocytes, and lymphocytes, as well as the crucial roles of Hb and albumin in tissue oxygenation and nutritional status, it might be possible to formulate some insightful remarks. Reduced peripheral lymphocyte counts insinuate a severely compromised immune response and intensified chronic systemic inflammation [40]. The production and activation of inflammatory chemokines and cytokines are heavily dependent on neutrophil and monocyte counts [41]. These cells may also inhibit T-cell activation and proliferation, thereby suppressing immune responses and exacerbating ongoing systemic inflammation [41]. The systemic inflammation response index (SIRI) is created by combining the three cellular elements of the H-Index. So, the H-Index can also be expressed as H-Index = [SIRI ÷ (Hb × Albumin)^−1^] × 100. Despite SIRI’s well-established prognostic value in patients with LA-NPC [42], there hasn’t been any prior research linking radiation-induced toxicity and pretreatment levels in LA-NPC or other head and neck cancers. However, Somay et al. recently showed that the SII, a variant of SIRI where only the monocytes are replaced by platelets in the formula, was related to worse TMJ arthrocentesis results [12]. Two more investigations by Somay et al. found that parotid gland cancer and LA-NPC patients with a high pre-radiotherapy neutrophil-to-lymphocyte ratio (NLR), a factor of the H-Index, and a low HPR had vastly higher rates of RIT [14, 15]. These data clearly show that elevated blood-borne cellular marker concentrations increase RIT occurrence and reduce treatment effectiveness after TMJ arthrocentesis.

Albumin and Hb are the non-cellular components of the H-Index formula. There is a universal consensus that damaged tissues need increased oxygenation [43], hence, low Hb levels may function as a systemic indirect surrogate signal for tissue hypoxia and poor tissue repair, such as the RIT and ORNJ. Marx’s hypoxic-hypocellular-hypovascular hypothesis of ORNJ lends some credence to this assertion [44]. According to this hypothesis, following radiation exposure, hypoxic, hypovascular, and hypocellular tissue develops, followed by a chronic, non-healing necrotic process caused by persistent hypoxia. By elevating TGF-beta, VEGF, and CD-31 (an endothelial cell marker), radiation-induced hypoxia may worsen an already existing hypoxic state and accelerate late tissue harm [45]. This finding suggests that the fibrinogenic and angiogenic pathways are crucial in radiation-induced late tissue injuries like the RIT and ORNJ. According to fibroatrophic theory, RIT and ORNJ may be caused by TMJ, mandibular elevator muscle, and jaw fibrosis [46, 47]. Cancer patients often have hyper-catabolic, hyper-inflammatory, and malnourished states, which can inhibit albumin synthesis via elevated C-reactive protein [22, 23]. Thus, low albumin levels may indicate poor immunity, chronic inflammation, nutritional status, and muscle mass loss in such patients, including masticatory and vascular muscle layers. These data offer a strong foundation for the elevated risk of RIT and ORNJ in LA-NPC patients with a high H-Index, as observed here, even though the precise mechanism is probably more complex.

Biomarkers Definitions Working Group defines a biomarker as a characteristic that is objectively measured and evaluated as an indicator of normal biologic processes, pathogenic processes, or pharmacologic responses to a therapeutic intervention [48]. The following characteristics should be present in an ideal clinical biomarker according to Lesko and Atkinson [49]: [1] clinical relevance, [2] high sensitivity and specificity, [3] reliability, [4] practicality, and [5] simplicity. An ideal biomarker should also be replicable, simple to achieve and perform, and affordable enough to be widely adopted. Given these features and the consistent findings from H-Index studies and those presented here, the H-Index appears to be an excellent biomarker for predicting survival outcomes and severe late toxicities in LA-NPC patients.

The present study is strengthened by several factors. First, head and neck MRI and PET-CT were the standard initial staging procedures for all qualified patients to improve NPC staging, target volume delineation, and response and toxicity assessments. Second, the unexpected biasing effect of disease and treatment variables may have been reduced because all patients had a comparable disease stage and underwent a standard oral examination and C-CRT protocol. Third, all patients had their H-Index constituents measured on the first day of C-CRT, which may have mitigated the effects of time-dependent parameter variations. However, this study has some drawbacks. First, since the results presented here would have been unintentionally biased in favor of one group by some unforeseen factors, a problem bedeviling all retrospectively designed single-institutional investigations, they should only be considered hypothesis-generating. Second, the H-Index cutoffs used here and their effects on the results reflect only a single time-point estimation and related RIT and ORNJ rates that skip the fluctuating nature of the measures of the H-Index constituents. Thus, future comprehensive research on H-Index dynamics may provide more dedicated cutoff(s) for more accurate RIT and ORNJ rate prediction. And third, without the H-Index and cytokine/chemokine correlations, we may have missed the chance to assess and offer insights into possible mechanistic links between the H-Index and other nutritional and immune-inflammatory factors.

## Conclusion

The current study examined the H-Index’s ability to predict RIT and ORNJ rates in LA-NPC patients receiving definitive C-CRT. Our findings demonstrated that using a cutoff value of 5.5, the RIT and ORNJ rates after C-CRT could be effectively separated into two risk groups If corroborated by further research, these results might help stratify the risk of these individuals and build stricter follow-up algorithms for high-risk populations.

## Data Availability

For researchers who satisfy the criteria for access to sensitive data, the datasets utilized and/or analyzed during the current study are accessible from the Baskent University Department of Radiation Oncology Institutional Data Access: adanabaskent@baskent.edu.tr.
